# Binding of Catechins to Staphylococcal Enterotoxin A

**DOI:** 10.3390/molecules23051125

**Published:** 2018-05-09

**Authors:** Yuko Shimamura, Mio Utsumi, Chikako Hirai, Shogo Nakano, Sohei Ito, Ai Tsuji, Takeshi Ishii, Takahiro Hosoya, Toshiyuki Kan, Norio Ohashi, Shuichi Masuda

**Affiliations:** 1 School of Food and Nutritional Sciences, University of Shizuoka, 52-1 Yada, Suruga-ku, Shizuoka 422-8526, Japan; shimamura@u-shizuoka-ken.ac.jp (Y.S.); s16204@u-shizuoka-ken.ac.jp (M.U.); gp1848@u-shizuoka-ken.ac.jp (C.H.); snakano@u-shizuoka-ken.ac.jp (S.N.); itosohei@u-shizuoka-ken.ac.jp (S.I.); ohashi@u-shizuoka-ken.ac.jp (N.O.); 2 Faculty of Nutrition, Kobe Gakuin University, 518 Arise, Ikawadani-cho, Nishi-ku, Kobe 651-2180, Japan; atsuji@cc.nara-wu.ac.jp (A.T.); ishii_t@nutr.kobegakuin.ac.jp (T.I.); 3 Department of Kampo Pharmacy, Yokohama University of Pharmacy, 601 Matano-cho, Totsuka-ku, Yokohama 245-0066, Japan; hosoya@toyo.jp; 4 Department of Synthetic Organic & Medicinal Chemistry, School of Pharmaceutical Sciences, University of Shizuoka, 52-1 Yada, Suruga-ku, Shizuoka 422-8526, Japan; kant@u-shizuoka-ken.ac.jp

**Keywords:** staphylococcal enterotoxin A, catechins, (−)-epigallocatechin gallate, molecular docking

## Abstract

Staphylococcal enterotoxin A (SEA) is a toxin protein, and is the most common cause of staphylococcal food poisoning. Polyphenols, such as catechins, are known to interact with proteins. In this study, we investigated the binding of catechins to SEA using SPR (Biacore), Fourier transform infrared spectroscopy (FT-IR), isothermal titration calorimetry (ITC), and protein-ligand docking. We found that (−)-epigallocatechin gallate (EGCG) could strongly bind to SEA. According to thermodynamic parameters, a negative Δ*G* indicated that the interaction between EGCG and SEA was spontaneous, and the electrostatic force accompanied by hydrophobic binding forces may play a major role in the binding. Data from Western blot analysis and docking simulation suggest that the hydroxyl group at position 3 of the galloyl group in the catechin structure was responsible for binding affinity with the Y91 of the A-6 region of SEA active sites. Our results provide further understanding of the binding interactions between catechins and SEA, and the inhibition of toxin activities by catechins.

## 1. Introduction

*Staphylococcus aureus* (*S. aureus*) produces the protein toxin staphylococcal enterotoxin (SEs), causing staphylococcal food poisoning [1]. Among SEs, more than 80% of staphylococcal food poisoning is caused by staphylococcal enterotoxin A (SEA) [2]. SEA has superantigenic activity as well as emetic activity, causing toxic shock syndrome due to a massive release of cytokines [3]. It has been reported that peptide regions of SEA fragments A-2, A-3, and A-6 (21–40, 35–50, and 81–100 of SEA) were important for both superantigenic and emetic activities of SEA molecules, while A-10 (region 9 161–180) was involved in the superantigenic activity, but not emetic activity of the SEA [4]. The superantigen binds to the major histocompatibility antigen (MHC) class II molecule on the antigen presenting cell and activates the T cells via the T cell receptor (TCR). The superantigen causes excessive irritation to the immune system to abnormally proliferate T cells, and is involved in the onset or refractory of multiple sclerosis [5], rheumatoid arthritis [6], psoriasis, atopic dermatitis [7], and chronic rhinosinusitis [8]. Accordingly, the various diseases described above are developed by the production of a large amount of cytokine induced by SEA. Therefore, it is expected that these diseases can be alleviated by suppressing the superantigen activity of SEA.

Polyphenols are known to exhibit various physiological functions in vivo due to their structural diversity. Among them, green tea leaves contain a special class of bioflavonoids: catechin polyphenols [9]. The catechins in green tea have been characterized as (−)-epicatechin (EC), (−)-epigallocatechin (EGC), (−)-epicatechin gallate (ECG), and (−)-epigallocatechin-3-gallate (EGCG). EGCG is the main catechin (comprising ~50% of the total catechins present in green tea) [10]. Polyphenols are also thought to be likely to interact with proteins due to the structural factors of phenolic hydroxyl groups such as their hydrophobicity and hydrogen bonding properties. It has been reported that the binding of proteins and polyphenols produce a water-insoluble complex [11,12,13,14], and that polyphenols inhibit the toxin activity of staphylococcal proteins [15,16,17,18,19,20,21]. Although there is a report on the binding interaction analysis of staphylococcal enterotoxin B protein and polyphenols [22], there is no report on the binding interaction analysis of SEA proteins and polyphenols. Therefore, by clarifying the interaction between SEA and polyphenols, it is expected that important information can be obtained in discovering a control method of staphylococcal food poisoning.

The aim of our study is to demonstrate that catechins bind to SEA, and understand the structure–activity relationship between these selected catechins and their binding sites on the SEA molecule. In the present study, we investigated the binding interaction of catechins to SEA using Western blot analysis against active SEA toxin sites, and some physicochemical methods, such as surface plasmon resonance (SPR), Fourier transform infrared spectrometer (FT-IR), and isothermal titration calorimetry (ITC). Molecular docking studies were also used to assist in the understanding of the binding of catechins and SEA and the inhibition of catechins on SEA toxin activity. Physicochemical methods and molecular docking were performed to directly compare the predicted data with the experimental data from some methods, and determine the potential modes of action of catechins and SEA.

## 2. Results

### 2.1. Interaction between Catechins and SEA

**Figure 1:**
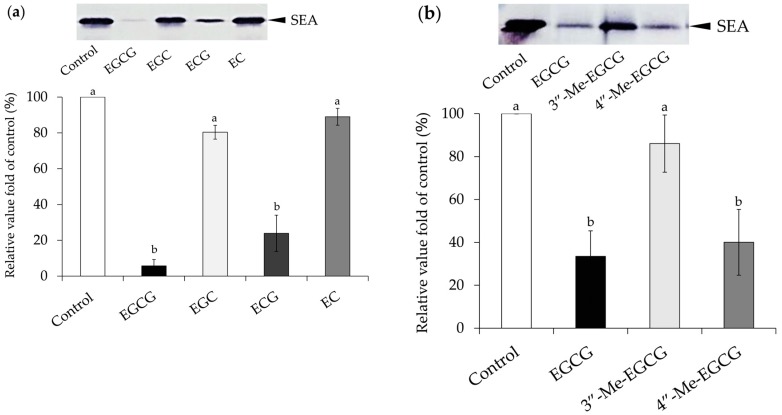
Interaction between catechins and staphylococcal enterotoxin A (SEA). (**a**) Interaction between four catechins and SEA; (**b**) Interaction between epigallocatechin gallate (EGCG) or methylated EGCG and SEA. Each catechin sample (**a**) and methylated EGCG (**b**) (final concentration 3.0 mM) and purified SEA (final concentration 100 ng/mL) was mixed and incubated at 37 °C for 24 h. After centrifugation, the supernatant was applied to SDS-PAGE and visualized by Western blot analysis with anti-SEA antibodies. MilliQ water (**a**) or dimethyl sulfoxide (**b**) was used as a positive control. The residual ratio of SEA protein was determined by Western blot analysis and quantified using ImageJ software (National Institutes of Health, Bethesda, MD, USA). There is a significant difference between different alphabets (Tukey–Kramer test, *p* < 0.05). Values represent the mean ± SD for three independent experiments.

The interaction between catechin and SEA was examined by Western blot analysis using anti-SEA polyclonal antibody. EC, ECG, EGC, EGCG, (−)-3″-Me-EGCG, and (−)-4″-Me-EGCG were tested at a final concentration of 3.0 mM (*p* < 0.05) (Figure 1). As a result, SEA bands were significantly decreased with the addition of EGCG, ECG (Figure 1a), and (−)-4″-Me-EGCG (Figure 1b), suggesting that they interact with SEA. Especially, EGCG strongly reduced the SEA band intensity. In contrast, SEA bands were found by treatment with EC, EGC (Figure 1a), and (−)-3″-Me-EGCG (Figure 1b). These results may mean that the hydroxyl group at position 3 of the galloyl group in the catechin structure was responsible for the binding affinity with the SEA.

### 2.2. Interaction between Catechins and SEA Toxin Active Sites

**Figure 2:**
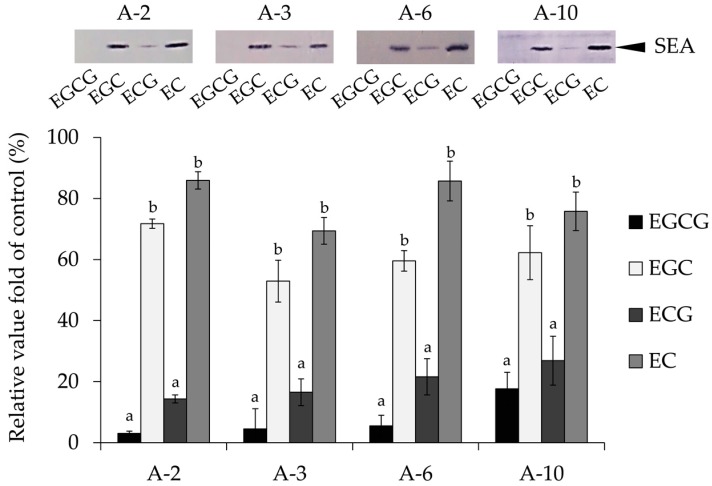
The interaction of catechins on SEA active sites. Each sample (final concentration of 3.0 mM) was mixed with SEA (final concentration of 5.0 μg/mL) and incubated at 37 °C for 24 h. After centrifugation, the supernatant was applied to SDS-PAGE and visualized by Western blot analysis with the anti-peptide of active SEA site antibodies. MilliQ water was used as a positive control. The residual ratio of SEA protein was determined by Western blot analysis and quantified using ImageJ software (National Institutes of Health, Bethesda, Maryland, United States of America). There are significant differences between different alphabets (Tukey–Kramer test, *p* < 0.05). Values represent the mean ± SD for three independent experiments.

After incubating catechins (EC, ECG, EGC, and EGCG; final concentration 3.0 mM) and SEA (final concentration 100 ng/mL), Western blot analysis was done using four different anti-peptide antibodies that bind to the A-2, A-3, A-6, and A-10 peptides corresponding to the regions 21–40, 35–50, 81–100, or 161–180 in active SEA toxin sites [4]. As a result, the SEA band was significantly decreased in EGCG and ECG at all active SEA toxin sites (Figure 2). Of these catechins, EGCG, in particular, strongly reduced the band intensity of anti-peptides of active SEA site antibodies.

### 2.3. Surface Plasmon Resonance (SPR) Sensor Measurements

**Figure 3:**
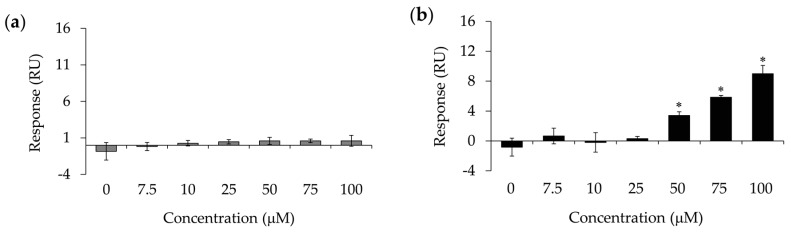
BIAcore analysis of the binding of catechins to SEA. (**a**) The binding of epicatechin (EC) to SEA; (**b**) The binding of EGCG to SEA. The affinity of catechins was examined based on surface resonance using a BIAcore 2000 biosensor instrument, with SEA immobilized on the flow cell of the sensor chip CM5. The binding responses are represented in resonance units (RU). A running buffer was used as a negative control (0 μM). * represents *p* < 0.05 compared to the control.

The binding affinity of EGCG, which reduced the band intensity of SEA and active SEA sites more than other catechins with SEA molecules, was determined using SPR on a Biacore 2000 instrument (GE Healthcare Bio-Sciences KK, Tokyo, Japan) (with SEA immobilized on the flow cell of the CM5 sensor chip. The immobilization amount of SEA on the sensor chip was 966.5 resonance units (RU). EC at a concentration of 100 µM did not react with SEA immobilized on the chip (Figure 3a). On the other hand, EGCG at a concentration of 50 µM or more was immediately reactive with SEA immobilized on the chip (Figure 3b).

### 2.4. Fourier Transform Infrared Spectroscopy (FT-IR) Measurements

**Figure 4:**
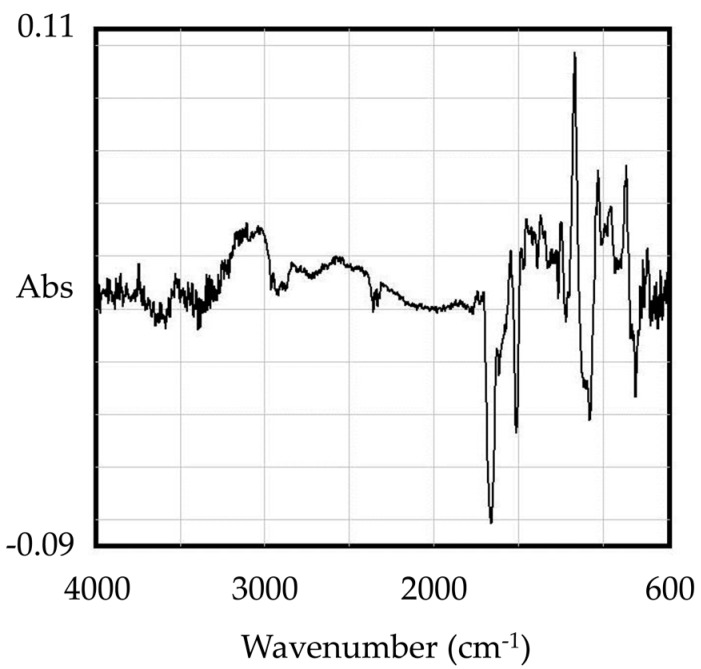
Difference spectrum between precipitation of SEA + EGCG and EGCG. The IR spectrum of EGCG was subtracted from the IR spectrum of precipitation of SEA + EGCG.

A difference spectrum was calculated from the IR spectrum of the precipitation of SEA + EGCG and EGCG. As a result, the spectra of EGCG-SEA are characterized by the typical aromatic double-bond stretching at about 1600 cm^−1^ (Figure 4).

### 2.5. Isothermal Titration Calorimetry (ITC) Measurements

**Figure 5:**
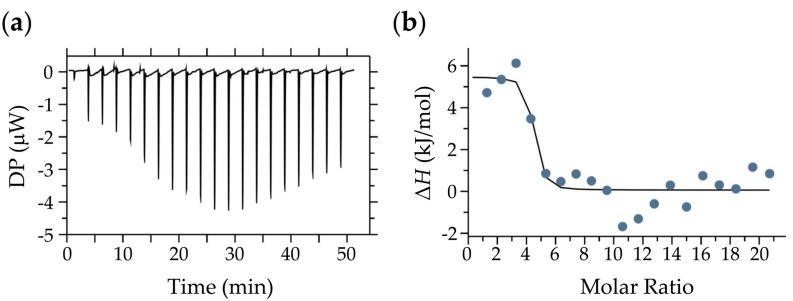
Isothermal titration calorimetry (ITC) thermogram and titration plots of EGCG into SEA. (**a**) Raw data plot of heat flow over time for the titration of 2.0 mM of EGCG into 20-µM SEA; (**b**) Corresponding plot after integration of peak areas and normalization to yield a plot of molar enthalpy change against the EGCG/SEA ratio. The one-site fit curve is displayed as a thin line.

Detection of thermodynamic parameters for catechin–SEA interaction by ITC provides valuable information about the binding form of catechin to SEA. The results of the ITC analysis of the data are shown in Table 1 along with the derived parameters (Δ*H*, Δ*S*, Δ*G*, and −*T*Δ*S*). The Δ*H* and Δ*G* values for EC–SEA interaction were −6.19 kJ mol^−1^ and −11.7 kJ mol^−1^, respectively. An interaction between EC–SEA was not detected (Table 1). Raw ITC profiles obtained by the titration of EGCG to the SEA solution and the corrected injection heat values are shown in Figure 5. The interaction between EGCG–SEA was exothermic. An enthalpy change (Δ*H*) of 5.41 kJ mol^−1^ and free energy change (Δ*G*) of −38.3 kJ mol^−1^ was obtained from the ITC data (Table 1). It was entropically driven based on Δ*H* > 0 and −*T*Δ*S* < 0. The interactions were accompanied by a large change in enthalpy, and a large negative value in Δ*G* represents a high affinity [23]. According to thermodynamic parameters, a negative Δ*G* indicated that the interaction between EGCG and SEA was spontaneous, and the electrostatic force accompanied by hydrophobic binding forces may play a major role in the binding.

### 2.6. Molecular Docking and Binding Site Analysis

**Figure 6:**
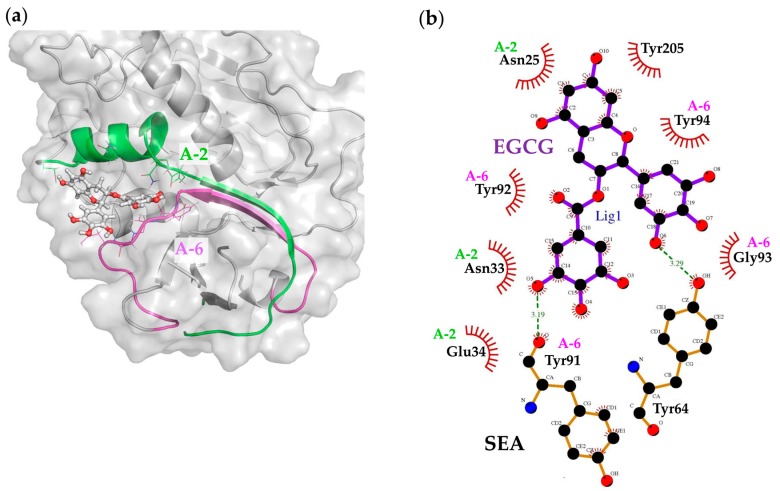
Docking model of staphylococcal enterotoxin A (SEA) protein–EGCG complex. (**a**) The binding site of EGCG on SEA. A-2 (corresponding to amino acid residues 21–40; green) and A-6 (corresponding to amino acid residues 81–100; magenta). (**b**) Interaction maps of EGCG with SEA. The binding affinity of EGCG to SEA protein was schematic to a two-dimensional (2D) image of protein-ligand complexes using LIGPLOT in silico.

**Figure 7:**
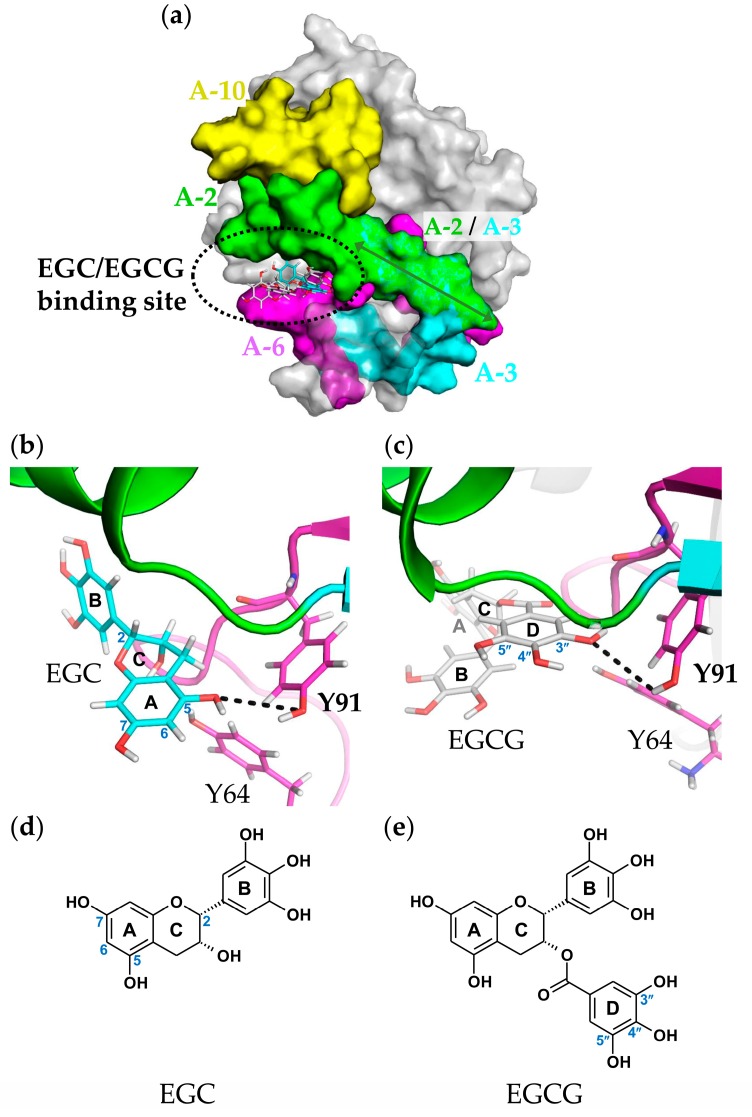
Autodock blind-docking of EGCG and epigallocatechin (EGC) to staphylococcal enterotoxin A (SEA) protein. (**a**) The binding site of EGC or EGCG on SEA. A-2 (corresponding to amino acid residues 21–40; green), A-3 (corresponding to amino acid residues 35–50; cyan), A-6 (corresponding to amino acid residues 81–100; magenta) and A-10 (corresponding to amino acid residues 161–180; magenta); (**b**) Docking simulation for the binding of EGC to the active sites of SEA (A-6 region); (**c**) Docking simulation for the binding of EGCG to the active sites of SEA (A-6 region); (**d**) The 2D structures of the EGC; (**e**) The second structures of the EGCG.

As a result of the docking simulation of EGCG and SEA, it was considered that EGCG bind to the pocket area created by A-2 (green) and A-6 region (magenda), as predicted by Western blot analysis with the anti-peptide of the active SEA site antibodies (Figure 6a). In addition, it was thought that Y91 (the A-6 region) of SEA formed a hydrogen bond to the 3″ position of the galloyl group of EGCG, and amino acid residues in the A-2–A-3 and A-6 regions formed a hydrophobic bond (Figure 6b). 3″-methylated EGCG exhibited a weaker binding force with SEA than EGCG (Figure 1b). Since 3″-methylated EGCG was methylated at the 3″ position of the galloyl group of the EGCG structure, hydrogen bonding with Y91 was broken, and the interaction was weakened. The docking simulation of EGC and SEA revealed that the binding site with SEA is common with EGCG, suggesting that the A-6 region forms an important interaction for binding with catechins (Figure 7a). However, in the EGC without the galloyl group, the A-ring was considered to bind to Y91 (A-6 region) (Figure 7b).

## 3. Discussion

The galloyl catechins such as ECG and EGCG bind more strongly than EC or EGC to phospholipids, proteins such as salivary proteins, milk proteins, caseins [24,25,26], and staphylococcal membranes [27], and penetrate deep into the hydrophobic core of the bacterial cytoplasmic membrane [12,24,28]. Hydrophobic bonds, due to the presence of galloyl groups, are thought to play a major role for higher affinity. Among the galloyl catechins, ECG has a greater affinity for the phospholipids than EGCG [13,29]. In the interaction between catechins and phospholipids, the presence of galloyl groups and the number of hydroxyl groups affect only the strength of hydrophobic bonds [25,26]. Although in the case of proteins, the hydrophobic bond by the galloyl group is the most influential factor for the interaction, the hydroxyl group in the B-ring also contributes to the hydrogen bonding with amino acid residues of the protein [13,14]. It has been reported that factors other than the hydrophobic bond (hydrogen bond, electrostatic interaction, and three-dimensional structure) are also involved in the interaction between catechins and proteins, and a change of affinity between proteins is expected [30,31].

In this study, the binding of catechins to SEA, a toxin protein most involved in the occurrence of staphylococcal food poisoning, was examined. EGCG has been shown to bind to proteins in saliva [32,33] as well as various proteins such as a 67-kDa laminin receptor in vivo [34]. Although various polyphenols with the action of binding and aggregating protein in foods have been reported [11,35], there has been little reported on polyphenols binding to SEA. Therefore, we have considered the binding of catechins, which are major components of green tea, to SEA for a further understanding of how these compounds may be efficiently used to prevent food poisoning.

Catechins (EGCG, EGC, ECG, EC, (−)-3″-Me-EGCG and (−)-4″-Me-EGCG) and SEA were incubated at 37 °C for 24 h, and the interaction was examined by Western blot analysis. As a result, it was suggested that EGCG, ECG (Figure 1a), and (−)-4″-Me-EGCG (Figure 1b) could interact with SEA. In (−)-3″-Me-EGCG, the interaction became weak, suggesting that the 3″ position of the galloyl group of EGCG or ECG is related to the interaction with SEA. Western blot analysis also examined the interaction between the SEA toxin active sites (A-2, A-3, A-6, and A-10) and catechins. As a result, EGCG and ECG interacted with all four toxin active sites (Figure 2). These results suggested that the presence of the galloyl group is involved in the interaction of the catechins with the site of toxin activity of SEA.

We analyzed the binding affinity of EGCG to SEA molecules using BIAcore analysis. As a result, EGCG was bound to SEA molecules immobilized on the chip in a dose-dependent manner (Figure 3). However, EC was not bound to the SEA molecules immobilized on the chip. These results suggest that the galloyl group reacts with SEA molecules, and EGCG may directly block the binding of the antibodies to the epitope of active SEA toxin sites.

From the IR spectrum of the precipitate obtained by the reaction of EGCG and SEA, a difference was observed in the absorption wavelength around 1600 cm^−1^ (Figure 4), and the energy state was changed by the binding of EGCG to SEA. Then, ITC was used to determine the thermodynamic properties of EGCG–SEA binding interaction. Thermodynamic parameters based on a −Δ*G* < 0, Δ*H* > 0, and −*T*Δ*S* < 0 indicated that the interaction between EGCG and SEA was entropically driven (Figure 5, Table 1). The explanation for the entropically driven reaction was the hydration effect during the complex formation, so that water molecules were released from the complex interface [36]. The negative Δ*G* meant that the binding process was a spontaneous biomolecular reaction. The binding enthalpies implied that the interaction was non-covalent, since the Δ*H* were low (5.41 J/mol) for covalent bond formation to have occurred (200–400 kJ/mol) [37]. These results suggested that the binding of EGCG to SEA was spontaneous, and the electrostatic force accompanied by hydrophobic binding forces may play a major role in the binding. It has been reported that although the interaction between (+)-catechin and β-casein was endothermic, the reaction was entropically driven [36]. In the future, it is necessary to examine the interaction with SEA by considering food-derived proteins such as milk casein.

As a result of the docking simulation, it was considered that EGCG is bound to the pocket created by A-2–A-3 (green) and the A-6 region (magenda) (Figure 6a). Since the results of the ITC analysis indicated hydrophobic bonding between EGCG and SEA, this finding is also supported. In addition, it was suggested that the 3″ position of the galloyl group of EGCG and the A ring of EGC interact with the Y91 of the A-6 region of active SEA sites (Figure 6b and Figure 7c). When both the galloyl group and the A-ring exist in the structure in catechins, it was considered that the galloyl group selectively interacts with SEA (Figure 7). There are nine hydrogen bonds in the SEA–T cell receptor (TCR) complex [38]. Among them, there are four regions of SEA involved in binding TCR: the 2-helix (residues 20, 21, 24, 25, 27, and 32–34), the hydrophobic patch consisting of the β2–β3 and β4–β5a loops (residues 62–64 and 91–94), the α4-β9 loop (residues 174 and 175), and the N-terminal side of the α5-helix (residues 205 and 206) [38]. It was suggested that Y91 of the A-6 region of active SEA sites and EGCG can form a hydrogen bond, suggesting that SEA-induced toxin activity might be inhibited.

## 4. Materials and Methods

### 4.1. Reagents

Staphylococcal enterotoxin A (greater than 95% purity; Toxin Technology, Sarasota, FL, USA) was used through all of the studies. (−)-Epicatechin (EC), (−)-Epicatechin gallate (ECG), (−)-Epigallocatechin (EGC), and (−)-Epigallocatechin gallate (EGCG) (Figure 1) were from Wako Pure Chemical (Osaka, Japan). All of the catechins used were of analytical grade. Synthesis of methylated EGCG ((−)-3″-Me-EGCG and (−)-4″-Me-EGCG) were performed according to our reported method [39].

### 4.2. Interaction between Catechins and SEA

Each catechin (EC, ECG, EGC, EGCG, and EGCG) and methylated EGCG ((−)-3″-Me-EGCG and (−)-4″-Me-EGCG) stock solutions (300 mM) was diluted in 10% dimethyl sulfoxide and dimethyl sulfoxide, respectively. Each catechin and methylated EGCG was diluted in MilliQ water and dimethyl sulfoxide, respectively. The interaction between each catechin and methylated EGCG (final concentration 3.0 mM) and purified SEA (final concentration 100 ng/mL) was estimated as previously described [19,20]. Each catechin and SEA were incubated at 37 °C for 24 h. After the reaction mixture was centrifuged (4000× *g*, 5 min), the resultant supernatant was used for SDS-PAGE (15% SDS-PAGE gel) and Western blot analysis. A Protein Detector Western Blotting Kit, BCIP/NBT System (KPL, Gaithersburg, MD, USA), was used to detect the SEA signals according to the manufacturer’s instructions. MilliQ water or dimethyl sulfoxide were performed as a positive control. The residual ratio of SEA protein was determined by Western blot analysis and quantified using ImageJ software (NIH, Bethesda, MD, USA).

### 4.3. Interaction between Catechins and SEA Toxin Active Sites

There are four SEA regions that are responsible for the superantigenic and emetic activities on SEA structures [4]. According to the amino acid sequence of SEA reported previously, these four peptides (A-2, A-3, A-6, and A-10, each 20 amino acid residues in length) corresponding to the fragment of the primary sequence of SEA were synthesized in detail previously [19]. A-2, A-3, A-6, and A-10 peptides correspond to the regions 21–40, 35–50, 81–100, or 161–180 in active SEA toxin sites [4]. Each catechin (EC, ECG, EGC, and EGCG; final concentration 3.0 mM) and SEA (final concentration 100 ng/mL) were incubated at 37 °C for 24 h. After the reaction mixture was centrifuged (4000× *g*, 5 min), the resultant supernatant was used for SDS-PAGE and Western blot analysis using four synthetic peptides equivalent to active SEA toxin sites, and rabbit antibodies to their corresponding peptides. A Protein Detector Western Blotting Kit, BCIP/NBT System (KPL, Gaithersburg, MD, USA), was used to detect the SEA active site signals according to the manufacturer’s instructions. SEA band intensity was indicated as the relative value when the SEA band intensity of the positive control is taken as 100%. MilliQ water was performed as a positive control. The residual ratio of SEA protein were determined by Western blot analysis and quantified using ImageJ software.

### 4.4. Surface Plasmon Resonance (SPR) Sensor Measurements

The binding affinity of EGCG to SEA was examined by SPR (BIAcore 2000 biosensor instrument; GE Healthcare Bio-Sciences KK, Tokyo, Japan). SEA (500 μg/mL in Borate 8.5 (10 mM borate buffer, pH 8.5; GE Healthcare Life Sciences) was immobilized on the flow cell of the CM5 carboxymethyl–dextran sensor chips using an amine coupling kit (GE Healthcare Life Sciences). EGCG were assayed over the immobilized SEA sensor surface at room temperature. EGCG (7.5 μM, 10 μM, 25 μM, 50 μM, 75 μM, and 100 μM) were injected and passed over the matrix-covered side of the flow cell. The flow rate was 20 μL/min, the binding time was three min, and the dissociation time was 10 min. After measurement, the sensor chip was reactivated (removal of residual bound substances) by adding 10 mM Glycine-HCl (pH 3.0) for 30 seconds. The interactions between the compound in the buffer solution and the immobilized molecules on the chip cause a change in refractive index in close proximity to the gold film surface that translates into a change in the resonance angle. The resonance angle is measured in resonance units (RU). One RU corresponds to approximately 10^−6^ refractive index units or approximately 1 pg/mm^2^ of adsorbed protein. A kinetic curve fitting was performed using BiaEvaluation software (version 3, GE Healthcare Bio-Sciences KK, Tokyo, Japan).

### 4.5. FT-IR Measurement

EGCG (final concentration 15 mM) and SEA (final concentration 25 µg/mL) were incubated at 37 °C for 4 h. After the reaction mixture was centrifuged (4000× *g*, 5 min), the resultant precipitation was lyophilized and used for FT-IR. The FT-IR spectrum sample was obtained using a FT/IR-550 spectrometer (JASCO Corporation, Tokyo, Japan) in the range 600–4000 cm^−1^ using the KBr method. A lyophilized product of the EGCG solution was performed as a control.

### 4.6. Isothermal Titration Calorimetry (ITC) Measurements

ITC was used to determine the thermodynamic properties of the EGCG–SEA binding interaction. ITC experiments were performed using a MicroCal PEAQ-ITC (Malvern Instruments Ltd., Malvern, Worchestershire, UK) thermostat at 37 °C. EGCG and SEA were suspended in phosphate-buffered saline (PBS; 0.1 M, pH 7.4), and the reference ampoule was filled with PBS solution. EGCG (2.0 mM) was placed in a 40-μL syringe, and 300 μL of SEA solution (20 μM) was placed in the sample cell. The EGCG solution was injected into the stirred sample cell containing SEA solution in 20 injections (2.0 μL/injection). To ensure proper mixing after each injection, a constant stirring speed of 750 rpm was maintained during the experiments. Control experiments were performed by injecting the ligand (EGCG) solution into the buffer, the buffer into the SEA protein solution, and the buffer into the buffer in an identical manner. The change in enthalpy (Δ*H*), the change in free energy (Δ*G*), and the change in entropy (Δ*S*) were determined using the manufacturer’s Microcal PEAQ-ITC analysis software. To compare with EGCG, the same concentration of EC solution was used.

### 4.7. Molecular Docking and Binding Site Analysis

The crystal structure of the SEA (PDB ID: 1ESF) was downloaded from RCSB PDB. The structure of SEA in chain A was utilized as a target of the docking simulation. Ligand parameter files for EGC or EGCG were prepared by downloading a mol2 file from the ZINC database [40]. The docking simulation was performed by submitting both SEA structure and ligand parameter files to SwissDock software (the Molecular Modeling Group of the Swiss Institute of Bioinformatics in Lausanne, Switzerland) [41]. In several of the docking structures generated by the simulation, only one structure was selected by referring to the results of the wet experiment in this study.

### 4.8. Statistical Analysis

The results were analyzed using a Student t-test or one-way ANOVA, followed by the Dunnett’s test using Microsoft Excel 2013 (Microsoft, Redmond, WA, USA). The significance level was set at *p* < 0.05 or < 0.01, and all of the experiments were replicated at least three times, except ITC analysis data of Figure 5 (two replicates). The Tukey–Kramer test was used to compare differences between groups (*p* < 0.05).

## Figures and Tables

**Table 1:** Thermodynamic parameters for the interaction of EGCG and EC with SEA obtained from ITC at 310 K and pH 7.40.

Sample		Δ*H* (J/mol)	Δ*S* (J/mol/deg)	Δ*G* (kJ/mol)	−*T*Δ*S* (kJ/mol)
Cell	Syringe				
SEA	EC	−61.9	−162.2	−11.7	50.3
	EGCG	5.41	140.9	−38.3	−43.7

Δ*H*: Enthalpy change, Δ*S*: Entropy change, Δ*G*: Gibbs free energy change, *T*: Absolute temperature.
